# Cardiovascular Complications of Chronic Opium Consumption: A Narrative Review Article

**Published:** 2019-12

**Authors:** Mojtaba ZIAEE, Reza HAJIZADEH, Arash KHORRAMI, Nariman SEPEHRVAND, Saeideh MOMTAZ, Samad GHAFFARI

**Affiliations:** 1. Medicinal Plants Research Center, Maragheh University of Medical Sciences, Maragheh, Iran; 2. Department of Cardiology, School of Medicine, Urmia University of Medical Sciences, Urmia, Iran; 3. Department of Medicine, University of Alberta, Edmonton, AB., Canada; 4. Medicinal Plants Research Center, Institute of Medicinal Plants, ACECR, Tehran, Iran; 5. Cardiovascular Research Center, Tabriz University of Medical Sciences, Tabriz, Iran

**Keywords:** Opium, Cardiovascular disease, Drug interactions, Metabolic effects, Mortality

## Abstract

Opiates are the second most prevalent abused illicit substance after cannabis in the world. The latest United Nations Office on Drugs and Crime (UNODC) report estimated 30% increment in opium cultivation worldwide. High prevalence of opium consumption in eastern countries may be due to the high availability and traditional misconceptions. Opium consumption has been linked to hypertension, diabetes mellitus, dyslipidemia, and coronary artery diseases (CAD). In this review, we will review the association between opium use, cardiovascular diseases, and clinical outcomes. The present evidence suggests that chronic opiate consumption may increase the risk of cardiovascular diseases and related mortality.

## Introduction

Poppy (*Papaver somniferum* L.) is one of the ancient plants that was cultivated for millennia and used for both medicinal and recreational purposes (1). Opium is a dark sticky or crumbly mass exuded from the ripening capsule of opium poppy and consists of several alkaloids including approximately twelve percent morphine with lesser amounts of noscapine, codeine, papaverine and thebaine (2).

**Fig. 1:**
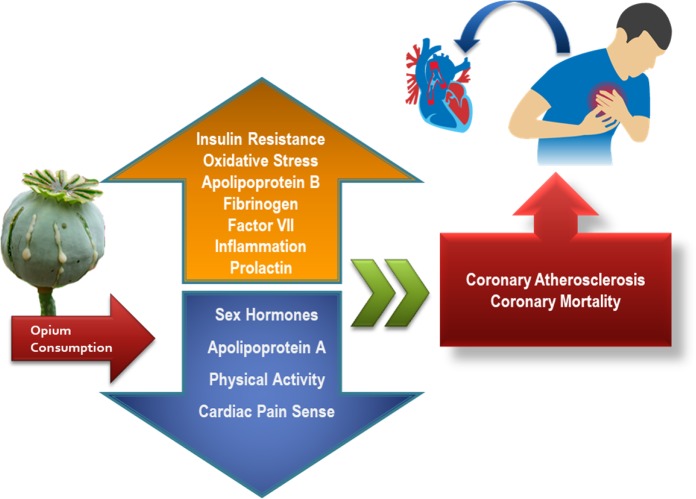
Adverse outcomes of opioids consumption

Raw opium is the second most prevalent abused substance after tobacco in most Asian countries. The 2017 report of the United Nations Office of Drugs and Crime (UNODC) estimated the overall production of opium to be 6,380 tons worldwide with 30% increase compared to the previous year and they reported the number of illicit users to be up to 17.7 million in 2015 (3). The high rate of opium consumption in Asian countries could be due to several factors such as widespread availability and long-standing misconceptions among ordinary people and even healthcare professionals regarding the purported alleviating effects of opium on coronary artery diseases (CAD), dyslipidemia, hypertension, and diabetes mellitus (DM) (4) (Fig. 1). Patients in the Middle East usually approach physicians with questions about the effectiveness of opium in controlling diabetes or cardiovascular conditions (5). The lack of sufficient evidence makes it difficult to answer those inquiries (6).

Recently opium has been proposed as a risk factor for cardiovascular diseases (CVD) (7–9). Tachycardia, bradycardia, and orthostatic hypotension are common cardiovascular problems seen in opium-addicted individuals (10). Plasma fibrinogen levels, coagulation, and atherosclerosis are adversely affected by opium abuse (11, 12). Low-density lipoprotein (LDL) cholesterol and triglyceride (TG) and blood glucose levels are other important factors changing in addicted people. They are known risk factors of CVD (13). Moreover, the deleterious effects of opium consumption on CVD risk factors were found to be proportional to the duration of consumption and the route of administration (14).

There is a paucity of consistent and reliable information on the association between opium dependency and CVD progression. Therefore, in the present article, we have reviewed the latest findings related to this issue.

### Pharmacology of Opiates

In 1806, Friedrich Wilhelm Sertürner isolated morphine as the active component of the opium poppy, and there the modern opioid pharmacology was born (15). More than 40 alkaloids exist in the milky latex fluid obtained from the opium poppy. The six major alkaloids that account for almost all of the natural alkaloid composition in opium are morphine, noscapine, codeine, papaverine, thebaine, and narceine (16). Thebaine is not used therapeutically, but several drugs such as naloxone, naltrexone, oxycodone, and buprenorphine are synthesized from thebaine.

The opioid receptors are categorized according to the International Union of Pharmacology (IUPHAR) recommendation to μ-(MOP), κ-(KOP), and δ-opioid (DOP) receptors, which are G-protein-coupled receptors (17). Cyclic AMP and/or ion channels (K+) are second messenger systems of opiate receptors. Studies suggested that modifications in the levels of cyclic AMP during chronic opiate consumption are associated with the development of tolerance and physical dependence (18).

### Interactions with cardiovascular medications

Opioid addicted patients may concurrently suffer from other comorbidities. Cardiovascular and pulmonary diseases are common among chronic opiate abusers but dose-dependent exact interactions are not studied well. Hence, the use of opiates (either therapeutic or on an abusive basis) along with cardiovascular medications (including anticoagulants, antiarrhythmic, cardiotonic, and antihypertensive drugs) may increase the risk of drug-drug interactions.

Concomitant administration of opiates with cardiovascular drugs may potentiate or reduce pharmacologic effects of cardiovascular medications such as warfarin or digoxin (19, 20). The interaction can affect the pharmacokinetics (absorption, distribution, metabolism or elimination) or pharmacodynamics (molecular mechanism of action) and the ultimate therapeutic status of the CV drugs (21, 22).

Opium contains alkaloids that have a direct impact on the cardiovascular or hemostatic systems. They may also have several indirect effects through interactions with the effect of other medications. Therefore, health care professionals should have a good knowledge base to identify possible opiate– drug interactions and to warn the patients about the possibility of complications. The lack of information on the interactions of opiates with concurrent medications needs to be addressed by well-designed clinical trials to assess the potential interactions and unknown side effects. Table 1 summarizes some important interactions of opiates with cardiovascular medications.

**Table 1:** Interactions between opiates and other medications

***Opiate***	***Medication***	***Extent of Interference***	***Mechanism***	***Comment***
Opium alkaloids	Isocarboxazide Linezolide Phenelazine Rasagiline Selegiline Tranylcypromine	Serious	Unknown	Risk of hypotension, hyperpyrexia, somnolence or death. Should separate by 2 weeks.(75)
	Procarbazine	Serious	Unknown	Potentiate CNS depression and hypotension(75)
	Fentanyl	Serious	Unknown	Risk of hypotension, respiratory depression and profound sedation, coma, death.(75)
	Amiodarone	Monitor closely	Cyp2d6	Prevents conversion of opiates to metabolites(75)
	Aspirin	Monitor closely	Unknown	Increase aspirin resistance(75 )
	CCBS	Monitor closely	Unknown	Potentiate effects of opiates(76)
	Dobutamine	Monitor closely	Unknown	Not clear
	Dopamine	Monitor closely	Unknown	Not clear
	Dronedarone	Monitor closely	Cyp2d6	Prevents conversion of opiates to metabolites
	Digoxin	Monitor closely	Unknown	Increase the risk of Digoxin toxicity(77)
	Ephedrine	Monitor closely	Unknown	Not clear
	Isoproterenol	Monitor closely	Unknown	Not clear
	Metaproterenol	Monitor closely	Unknown	Not clear
	Moxonidine	Monitor closely	Unknown	Not clear(75)
	Phenylephrine	Monitor closely	Unknown	Not clear
	Quinidine	Monitor closely	Cyp2d6	Prevents conversion of opiates to metabolites(75)
	Ticlopidine Ticagrelor(78) Clopidogrel(79) Prasugre(80)	Monitor closely	Cyp2d6	Prevents conversion of opiates to metabolites, decrease concentration and effects of ADP inhibitors
	Yohimbine	Monitor closely	Unknown	Not clear
	Warfarin and vk antagonists	Monitor closely	Unknown	Increase INR (81)

CCB: calcium channel blockers; CNS: Central nervous system; INR: international normalized ratio; PT: prothrombin time

### The effects of opium on CV risk factors

It seems that despite a few old studies, recent articles have emphasized the adverse effect of opium on cardiovascular risk factors. In the following sections, we will review the studies that explored the effect of opium consumption on cardiovascular risk factors such as hypertension, etc.

### Blood pressure and hypertension

There are misconceptions in the general population or even among some medical professionals, that opium could have favorable impacts on lowering blood pressure in hypertension while many experts may disagree. Opium use had no significant ameliorative effect on hypertension in either occasional or dependent users (9).

Similarly, several other studies failed to find a correlation between opium consumption and hypertension prevention (23–26). However, not all of these studies were consistent. A cohort study of 9,264 adults showed a lower prevalence of hypertension in opium users as compared to their counterparts, probably because of the younger age of opium users in that study population (27). In the contrary, in a cohort study on 5,332 participants, high systolic and diastolic blood pressures were more prevalent in opium users than in others (3). A case-control study reported the rate of hypertension to be significantly higher in addict patients with ischemic stroke than controls (28).

Endogenous opioid systems and opioid receptor agonists are purported to modulate the arterial pressure to some extent. Stimulation of peripheral opioid receptors may reduce arterial hypertension, especially in those with pronounced sympathetic hyperactivity or stress-based high blood pressure (29). Morphine administration could decrease systolic and diastolic blood pressures, however, in several other cases, morphine exposure enhanced hypertension through its effects on brain noradrenergic mechanisms or central opiate receptors (30–32). The dosage and duration of drug abuse seem to be critical factors here. Generally, short term and low dose exposure to opium lower blood pressure, whereas long-term dependence leads to hypertension. The former effect is attributed to vasodilation and opium's effect on reducing sympathetic tone. Opium-induced high blood pressure might be secondary to its long-term deleterious impact on the structure and function of body organs, particularly in the cardiovascular system, including microvascular coronary dysfunction, elevated plasma levels of homocysteine and fibrinogen, atheroma formation and related vascular stenosis (7, 11, 12). In general, data on opium-induced blood pressure alterations suffer from inadequacy and inconsistency, and further studies are warranted to shed some light on this issue.

### Effects on coronary disease and myocardial infarction

Opium users have shown to have a higher susceptibility to coronary artery disease compared to non-users with a dose-response relationship reported between these two (9, 33). Chronic opium consumers have an overall increase in their ECG abnormalities. The ECG abnormalities are more frequently observed in male opiate consumers than in females. QTc prolongation (13%), R and/or S wave abnormalities (11%), and poor R progression (10%) were the most reported ECG changes (34). Niaki et al. showed that opium consumption was a significant risk factor of MI with an adjusted odds ratio of 26.3. However, they did not find any association between opium abuse and extent of MI (35). In patients admitted with the diagnosis of MI the prevalence of opium addiction was 19%, while it was 2.8% in general population (36). Sadeghian et al. presented the opium abuse as a major risk factor for ischemic heart disease (8). In a study, they introduced diabetes mellitus in women and opium abuse in men as two major risk factors of CAD in Iran (37).

Hosseini et al. found a significant association between the dose of opium used by addicted patients and the Gensini score (β = 0.27, p = 0.04) (38). On the other hand, Dehghani et al. studied 239 opium addicts and 221 non-addicts with first MI and reported a lower rate of anterior wall MI and lower related early mortality in addicted as compared to non-addicted patients (39). Killip class and left ventricular ejection fraction (LVEF) were similar between addicted and non-addicted groups (39). None of the oral or inhaled routes of opium abuse have increased the occurrence of CAD in multivariate analysis (39).

Patients with MI should receive medical care promptly. Some addicted patients use opium to get relief from chest pain (41). Opium uses by relieving chest pain and inducing drowsiness can increase the time elapsed from symptom onset to admission to the hospital. This delay increases the mortality among opium users. Nevertheless, Bafghi et al. reported similar rates of chest pain between opium users and non-users, and hence, they attributed this delay in seeking medical care to other factors such as socioeconomic issues (36).

### Effect on heart failure

Heart failure and functional class seem to be no different in opium-addicted patients after MI than in non-addicted individuals. Davoodi et al. reported no difference between addicted and non-addicted patients regarding functional class, angiographic findings, and the need for CABG (42). Similarly, post-MI LVEF was not different between addicted and non-addicted patients (p = 0.4) (43). Safaei also reported no difference between the LVEF of addicted and non-addicted patients before and 6 months after CABG (44).

The effect of opium in patients with heart failure (HF) is still unclear. Morphine can lower some of the symptoms occurring in the late stages of HF such as dyspnea (45). Similarly, morphine seems to relieve the ischemia symptoms in patients with cardiovascular risk factors (46). The underlying pharmacological mechanism of morphine has encouraged some of the researchers to conclude that morphine may be cardioprotective in HF patients (47). However, one study revealed that patients with HF who use morphine along with nitroglycerine and furosemide had higher mortality rates (48). However, there is no enough data to provide evidence-based recommendations regarding the cessation of opium abuse in cardiac patients (49).

### Effects on cerebrovascular disease and stroke

A few studies assessed the effect of chronic opium consumption on the development of ischemic stroke. A case-control study showed a significant rise in ischemic stroke in opium-addicted patients in Kerman (*P* < 0.0001) (50). In another cross-sectional sonographic study conducted on 97 patients with ischemic stroke shown that there was no significant difference in the frequency of atherosclerosis and the type of involved vessels among opium addicts and non-addict patients (51). Rezvani and Ghandehari studied 558 opium users with a mean age of 56.2 years. They claimed that opium inhalation did not have a significant effect on occurrence cerebrovascular events and they concluded that opium may have a protective effect on ischemic stroke (40). Khademi et al in Golestan cohort study conducted on 5000 Iranian adults demonstrated that chronic abuse of opium was associated with an increased risk of ischaemic heart disease and cerebrovascular events (52). A recent case-control study presented opium addiction and hypertension as two independent predictors of stroke. After adjusting for tobacco smoking, opium abuse increased the risk of stroke by 2.3-fold (28).

### Effect on other serum parameters

In the majority of studies, opium addicts had higher levels of potassium (53–56), whilst opium consumption had either no or enhancing the effect on sodium levels (53, 55). Fe^2+^ levels were higher and total iron-binding capacity was lower in addicted diabetics as compared to non-addict diabetics (54). Albumin was lower among opium addicts; however, no significant difference was observed in albumin/globulin ratios (54). Urea and creatinine levels were enhanced by opium consumption in some studies (55, 57), but unchanged in others (58).

Serum transaminases are shown to be associated with the severity of coronary atherosclerosis, and both cardiovascular and all-cause mortality (4). Radmard et al conducted a cohort study on 1599 participants demonstrated a significant reduction in aspartate transaminase (AST) and alanine transaminase (ALT) in normal addicted people, while a statistical notable elevation in alkaline phosphatase (ALP) and gamma-glutamyl transferase (GGT) (59). As well as Karam et al. reported lower aspartate transaminase (AST) and alanine transaminase (ALT) in diabetic addicts as compared to non-addict diabetics (54). However, several animal and human studies have shown the opium consumption to be associated with increased levels of AST, ALT, ALP, lactate dehydrogenase (LDH) and GGT (55 , 57, 60, 61).

Opium abuse could potentiate cardiometabolic risk factors such as apolipoprotein B/apolipoprotein A-I index, which is a strong predictor of cardiovascular death (62). Other cardiac biomarkers including factor VII, fibrinogen, and homocysteine could be changed by chronic opium consumption (63). These findings might explain the increased risk of MI or stroke in opium users.

### Complication related to opium contamination

Opium impurities can impose a great health risk to chronic opium consumers. Chemical compounds such as lead, arsenic, and thallium are frequently presented in opium extract in different amounts. Depending on the area of opium production, product’s appearance and rout of consumption, smugglers forge opium by adding other impurities to maximize their profit (64). The motive behind adding lead to opium by drug smugglers is to increase the product’s weight (65–69). This will enhance the risk of lead poisoning among opium consumers. Signs and Symptoms include abdominal pain, constipation, anorexia, anemia and nephropathy (67–70). Acute and chronic accumulation of lead in the body causes cardiac and vascular damages with potentially life-threatening consequences (69). The blood lead level was found to be higher in opium consumers than non-addict individuals (65, 66, 70), but not across all studies (69). Opium inhalation seems to cause more lead surge in addicted subjects than oral use (65). Iron and calcium levels may affect lead absorption and their serum level may confound the lead-mortality association (71).

### Opium consumption and clinical outcomes

Despite common misconceptions among opium consumers, there is a higher rate of mortality and morbidity in opium addicts than non-users. There is a paucity of data on this matter mainly due to the lack of comprehensive large-scale, long-term, controlled studies. In-hospital mortality was significantly higher in opium-addicted patients compared to non-users. Due to the pain masking properties of opium, drug users may delay one-hour longer than non-users since the onset of MI symptoms to reach the emergency department (36). Morphine therapy in acute de-compensated heart failure increased cardiac biomarker (troponin I) levels significantly and raised the rate of adverse events such as mechanical ventilation, intensive care unit (ICU) admissions, prolonged hospitalization, and death (72). Safaii et al. reported chronic opium consumption to be associated with rehospitalization with cardiac cause within 6 months after CABG surgery (73). Other studies found no association between opium use and in-hospital mortality (39, 75), but it should be noted that these studies suffered from limitations such as small sample size and short-term follow-up periods.

## Conclusion

The incremental rise of opioid abuse requires us to inform and educate the patients regarding its possible detrimental effects. Several large-scale studies imply that opium is hazardous cardiovascular and metabolic disorders and even may worsen these complications.

Successive data demonstrated that there is an association between habitual opium consumption and the risk of ischemic events. While vernacular wrong believes and short-term and small-sized studies accentuate the usefulness of opium consumption. Therefore, clinicians and patients should be noticed about the deleterious effects of opium addiction on various vascular events. Besides these, further studies are warranted to elucidate the effects of opium use in cardiovascular conditions. Nevertheless, the existing data points to opium consumption as a risk factor of CAD and hence, strategies should be developed and implemented for the prevention and cessation of opioid abuse in the at-risk population.

## Ethical considerations

Ethical issues (Including plagiarism, informed consent, misconduct, data fabrication and/or falsification, double publication and/or submission, redundancy, etc.) have been completely observed by the authors.
